# Bioinformatics analysis combined with experiments to explore potential prognostic factors for pancreatic cancer

**DOI:** 10.1186/s12935-020-01474-7

**Published:** 2020-08-08

**Authors:** Mu-jing Ke, Lian-dong Ji, Yi-xiong Li

**Affiliations:** 1 Department of Ultrasound, Xiangya Hospital, Central South University, Changsha, 410008 Hunan People’s Republic of China; 2 Department of General Surgery, Xiangya Hospital, Central South University, Changsha, 410008 Hunan People’s Republic of China

**Keywords:** GEO, Pancreatic cancer, DLGAP5, Biological behavior

## Abstract

**Background:**

Pancreatic cancer is a common malignant tumor of the digestive tract. It has a high degree of malignancy and poor prognosis. Finding effective molecular markers has great significance for pancreatic cancer diagnosis and treatment. This study aimed to investigate DLGAP5 expression in pancreatic cancer and explore the possible mechanisms and clinical value of DLGAP5 in tumorigenesis and tumor development.

**Methods:**

Differentially expressed genes were screened using the Gene Expression Omnibus (GEO) data set GSE16515. Gene Ontology (GO)-based functional analysis and Kyoto Encyclopedia of Genes and Genomes (KEGG) pathways enrichment analysis were performed on the corresponding proteins of the above genes using the Database for Annotation, Visualization, and Integrated Discovery (DAVID). The Kaplan–Meier Plotter database was used to analyze the relationship between differentially expressed genes and pancreatic cancer prognosis. The most prognostic gene, *DLGAP5*, was screened out, and the Oncomine and gene expression profiling interactive analysis (GEPIA) databases were used to analyze its expression in pancreatic cancer and other cancer tissues. The Cancer Genome Atlas (TCGA) database was used to analyze the overall survival of DLGAP5. Gene set enrichment analysis (GSEA) was performed to explore its possible molecular mechanisms in pancreatic cancer. Furthermore, the biological behavior of DLGAP5 in pancreatic cancer was verified by cell function experiments.

**Results:**

A total of 201 significant upregulated differentially expressed genes and 79 downregulated genes were selected. The biological processes with significant enrichment of differential genes included cell adhesion, apoptosis, wound healing, leukocyte migration, angiogenesis. Pathways were mainly enriched in tumor-related signaling pathways such as cancer pathways, the extracellular matrix-receptor interaction pathway, and the p53 signaling pathway. DLGAP5 was significantly expressed in pancreatic cancer, and its expression level had a significant effect on patients’ survival time and progression-free survival. GSEA results indicated that *DLGAP5* had significantly enriched into signaling pathways such as the cell cycle, the p53 signaling pathway, and oocyte meiosis. The experimental results showed that when we knocked down the expression of *DLGAP5* in pancreatic cancer cells, their proliferation ability was significantly inhibited, and their invasion and migration ability significantly decreased.

**Conclusions:**

DLGAP5 can be used as a prognostic indicator for pancreatic cancer and affect the occurrence and development of pancreatic cancer.

## Background

Pancreatic cancer is a common malignant tumor of the digestive tract that is characterized by insidious onset, a high degree of malignancy, and rapid development [1]. More than 0.2 million people die from pancreatic cancer every year, and in Western countries, pancreatic cancer is the fourth most likely malignant tumor to cause death [2, 3]. Pancreatic cancer prognosis is poor, with a 5-year survival rate of 8% [4]. The incidence and mortality of pancreatic cancer has been increasing year by year worldwide, and 80% of patients have been found to have local metastasis when diagnosed [5]. Surgery is the main treatment for pancreatic cancer; however, 80% of patients are not suitable for surgery, and the recurrence rate among patients who have undergone resection is very high. Further, there are numerous postoperative complications, all of which result in unsatisfying surgical outcomes [6]. In recent years, research on molecular diagnosis and targeted biological therapy of disease has seen certain progress. Diagnosis and therapy have gradually become important means to improve the prognoses of patients with malignant tumors. Thanks to the rapid development of genomics, tumor-related genes are continuously being discovered. A number of research studies have shown that genes play vital roles in the incidence and development of pancreatic cancer [7–11].

The DLGAP protein family, which was originally detected in rats, consists of 5 members (DLGAP1, DLGAP2, DLGAP3, DLGAP4, and DLGAP5) distributed on different chromosomes to produce transcript variants of varying length [12, 13]. All DLGAPs share 3 key domains: a guanylate kinase-associated protein homologous structure, a 14-amino acid repeat domain, and a dynein light chain domain [14–16]. These specific regions enable DLGAP5 to interact with other proteins. DLGAP5 is a mitotic spindle protein that is thought to be a target of cell cycle controllers and Aurora kinase A. It can promote tubulin polymer formation, resulting in tubulin fragment production at the ends of microtubules. The consumption of DLGAP5 can lead to cycle prolongation and abnormal chromatin separation [17, 18]. DLGAP5 has been identified as a significant diagnostic and prognostic biomarker in human lung cancer, and silencing *DLGAP5* can considerably inhibit the proliferation and invasion of liver cancer cells [19, 20].

To the best of our knowledge, no study on *DLGAP5* in pancreatic cancer has been reported. Hence, this study was designed to investigate the role of *DLGAP5* as a biomarker in pancreatic cancer and explore the possible underlying mechanisms of *DLGAP5* in tumorigenesis. The target gene, *DLGAP5*, was screened out by the integrated Gene Expression Omnibus (GEO), Oncomine, and Gene Expression Profiling Interactive Analysis (GEPIA) databases. DLGAP5 was found to be differentially expressed in pancreatic cancer and related to prognosis. Further, we performed experiments to explore its molecular mechanisms in the development of pancreatic cancer, as it may serve as a prognostic marker for pancreatic cancer.

## Materials and methods

### Bioinformatics analysis

#### Selecting differential genes from the GEO database

The pancreatic cancer data set GSE16515 was obtained from the GEO. The data set contained 16 normal pancreatic tissue samples and 36 pancreatic cancer tissue samples. The platform and matrix files were downloaded. The R limma package was used to process the files, and then the data in the files were calibrated, standardized, and converted to a log2 scale. Differentially expressed genes (DEGs) were screened with the adjusted P-value of < 0.01 and |log fold change| of ≥ 2.

#### Construction of the protein–protein interaction network and screening of hub modules

To detect the potential relationships among DEGs, all the DEGs were mapped into the Search Tool for the Retrieval of Interacting Genes/Proteins (STRING) database. A confidence score ≥ 0.4 was set as the cut-off criterion. The cytoHubba [21] software was used to visualize the network. The Molecular Complex Detection (MCODE) algorithm was used to screen modules of the protein–protein interaction (PPI) network with a degree cut-off of 2, a node score cut-off of 0.2, a k-core of 2, and a maximum depth of 100. The Database for Annotation, Visualization, and Integrated Discovery (DAVID) Bioinformatics Resources (http://david.abcc.ncifcrf.gov/) were applied to perform Gene Ontology (GO)-based functional analysis for the corresponding genes of the proteins. Each PPI module was performed by applying DAVID. The steps were: paste the gene in “Enter gene list”; select the identifier “OFFICIAL_GENE_SYMBOL”; select the list type “Gene List”; and select “Go oncology” and “KEGG pathway” for analysis. P < 0.05 was set as the cut-off criterion.

#### Data analysis from Oncomine

Oncomine is a gene chip-based database and integrated data mining platform. In this database, the screening and data-mining conditions can be set according to users’ specific needs. The screening conditions set in this study were: (1) cancer type: pancreatic cancer; (2) gene: DLGAP5; (3) analysis type: cancer vs normal analysis; and (4) threshold conditions of P < 0.01, fold change > 2, and gene rank = top 10%.

#### Survival analysis by Kaplan–Meier plotter

An online survival analysis was performed using the pancreatic cancer data set from Kaplan–Meier plotter (http://kmplot.com/analysis/). The screening conditions were as follows: (1) cancer: pancreatic cancer; (2) gene: *DLGAP5*; (3) survival: OS/PFS; and (4) follow-up threshold: 80 months.

#### Differential expression analysis by GEPIA

GEPIA (http://gepia.cancer-pku.cn/) is a newly developed interactive web server for analyzing RNA sequencing expression data from the 9736 tumor samples and 8587 normal samples of The Cancer Genome Atlas (TCGA). The screening conditions were: (1) datasets selection: pancreatic cancer; (2) gene: *DLGAP5*; (3) expression DIY: boxplot; (4) cutoffs: P < 0.01 and fold change > 2; and (5) matched normal data: match TCGA normal and GTEx data.

#### Data collection from the TCGA

Pancreatic cancer data sets were downloaded from the TCGA. The pre-processing of TCGA data included the following steps: (1) remove normal tissue sample data; and (2) remove genes with a fragments per kilobase million (FPKM) of 0 in the samples. A total of 181 tumor tissue samples were included. DLGAP5 expression was ranked from low to high according to the expression profile, and the samples were equally divided into 4 parts.

#### Gene set enrichment analysis

Analysis was performed using the Gene Set Enrichment Analysis (GSEA) software (version 3.0). First, the “c2.cp.kegg.v6.1.symbols.gmt” data set was downloaded from the MsigDB database of the GSEA website. Second, the high-to-low grouped expression profile data and the attribute files were enriched and analyzed by default weighted enrichment statistics. The number of times of random combinations was set to 1000.

### Biological behavior experiments

#### Main reagents and equipment

Roswell Park Memorial Institute (RPMI) 1640 medium was purchased from Gibco/Thermo Fisher Scientific, Inc. Fetal bovine serum was obtained from Biological Industries. Mouse anti-p53 monoclonal antibody (catalog no. 2524S), rabbit anti-phospho-p53 polyclonal antibody (catalog no. 2521S), and rabbit anti-p21 monoclonal antibody (catalog no. 2947S) were purchased from Cell Signaling Technology, Inc. Rabbit anti-DLGAP5 polyclonal antibody (catalog no. PA5-82197) was purchased from Invitrogen Company. Mouse anti-actin monoclonal antibody (catalog no. sc-47778) was purchased from Santa Company. Every assay was done in triplicate.

#### Cell culture

The human pancreatic cancer cell lines PANC-1, SW1990, Capan-2, and BxPC-3 were cultured in RPMI 1640 medium containing 10% fetal bovine serum, which was placed in an incubator at 37 °C and 5% CO_2_. After 2 to 3 days of cell passage, the cells in the logarithmic growth phase were selected for further experiment.

#### Real-time polymerase chain reaction

The cell culture dish was placed on an ice plate after the culture liquid was discarded. The RNA was then extracted using the Trizol kit. The extracted RNA was reverse transcribed into cDNA per the flow of the PrimeScript^®^ RT reagent kit with gDNA Eraser (Shiga, Japan). The real-time quantitative polymerase chain reaction (RT-qPCR) reaction liquid was configured according to the flow of the SYBR^®^ Premix EX TaqTM II (Tli RNase H Plus; Shiga, Japan). The RT-qPCR was detected using the AppliedBiosystems^®^ 7500 Real-Time PCR Systems (ThermoFisher Scientific, Hampton, NH, USA), with 18S as the internal reference gene. The primer sequences for DLGAP5 were forward (5′–GAC AGG ATG CAG AAG GAG ATT ACT–3′) and reverse (5′–TGA TCC ACA TCT GCT GGA AGGT–3′). The 18S primer sequences were forward (5′–GGT GAA GGT CGG AGT CAA CGG–3′) and reverse (5′–GAG GTC AAT GAA GGG GTC ATTG–3′). Relative expressions were calculated using the 2^−∆∆Ct^ method.

#### Western blot experiment

The protein sample was cracked with 4 °C cracking buffer solution RIPA (1% Triton X-100, 50 mM Tris–HCl pH 7.4, 150 mM NaCl, 10 mM EDTA, 100 mM NaF, 1 mM Na3VO4, 1 mM PMSF, and 2 µg/ml Aprotinin) for 40 min. The sample was centrifuged at 13,000 rpm for 25 min. The supernatant was taken, and the protein was quantified by the Coomassie Brilliant Blue method. After mixing with 3 × sample buffer solution, it was boiled for 5 min. The sample (30–50 μg/lane) underwent electrophoresis in 12% SDS- polypropylene gel for 3 h and was then transferred to a nitrocellulose membrane (voltage: 2 mV/cm^2^; time: 120 min). After the sample was sealed with 5% skim milk for 1 h, the transfer film was clipped according to the molecular weight marked by the pre-stained marker. Primary antibodies (1:1000) were then added. The temperature was maintained at 4 °C overnight. After being washed 4 times with TTBS, secondary antibodies (1:2000) were added and kept at room temperature for 30 min. The sample was again washed with TTBS 4 times after using the enhanced chemiluminescence method to display color.

#### Cell transfection

The cells with good growth status and in the logarithmic growing phase were selected and digested into a single cell suspension and inoculated in a 6-hole cell crawling plate. The 6-hole cell plate was placed in an incubator for the night. After being adherent to the wall, the cells were prepared to be transfected with DLGAP5-siRNA and NC-siRNA at room temperature. A mixture of 5 μl lipofectamine 2000 was added to the RPMI 1640 medium and then set aside for 5 min. Again, 10 μl of siRNA was added to 240 μl of the RPMI 1640 medium and mixed with the lipo2000 prepared in the previous step. It was then placed still for 20 min. The original medium in the 6-hole cell plate was removed, and 1.5 ml of the RPMI 1640 medium was added to each hole. The configured transfection solution was then added and continued to be cultured in the incubator. After 6 to 8 h of transfection, the normal 1640 medium-containing serum was replaced.

#### MTT cell viability experiment

An MTT assay was used to measure cell viability. After being adherent to the wall, the cells were transfected with DLGAP5-siRNA and NC-siRNA. To evaluate cell viability, the absorbance values were detected before and 1 to 4 days after transfection. The specific operation was done by adding 20 μl of MTT solution (5 mg/ml) to each well in the 96-well culture plates and incubating for another 4 h. We removed the supernatant, added 200 μl of DMSO to each well in the 96-well culture plates, and oscillated to dissolve the crystalline matter. The absorbance was measured at 570 nm using a microplate reader (Model 550; Bio-Rad Laboratories, Inc., Hercules, CA, USA).

#### Colony formation experiment

Forty-eight hours after transfection, the cells were inoculated to a 12-hole culture plate and incubated in an incubator. The growth status of the cells was observed every 3 days. After 2 weeks, the colonies were fixed with formaldehyde and stained with 0.5% crystalline purple. The number of colonies was calculated.

#### Transwell experiment

The cells were digested, and the samples were centrifuged. After taking the supernatant and re-suspension with the RPMI culture solution, the sample was centrifuged and rinsed again. It was then re-suspended with the RPMI medium. The cell concentration was calculated, and the cells were configured into 200 μl of an RPMI culture medium cell suspension, mixed, and placed in the upper chamber of a microporous filter membrane with an 8-μm diameter. In the lower chamber of the Transwell, 500 μl of the 1640 medium containing 10% fetal bovine serum was added and incubated at 37 °C. The cells were used to determine migration and invasion by penetrating the membranes and the matrix gel-coated membrane, respectively. After 24 h, we removed the chamber, wiped off the remaining cells with a cotton swab, and dried it at room temperature. The sample was fixed with 4% paraformaldehyde and dyed for 1 min according to the Wright stain method. The sample was mixed with diluted Giemsa stain and re-dyed for 40 min. The filter membrane was dried with a cotton swab, and the sample was photographed.

#### Wound healing assay

The cells were selected and counted 2.0 × 10^5^ after digestion. After transfection with DLGAP5-siRNA and NC-siRNA for 48 h, when the cells grew to nearly 100% confluence, the monolayer cells were scratched with the tip of a 200-μl pipette and photographed using an inverted microscope. The 6-hole culture plate was replaced in the incubator and photographed again 24 h later.

#### Flow cytometry

The effect of DLGAP5-siRNA on apoptosis was detected. The cells to be treated were digested with trypsin, centrifuged, washed in phosphate-buffered saline (PBS), and suspended in 200 μl of buffer solution. Subsequently, 5 μl of Annexin V-FITC (BD Biosciences, Franklin Lakes, NJ, USA) was added to the 195 μl of cell suspension. After full mixing and incubation at room temperature for 10 min, the cells were washed with 200 μl of buffer solution and re-suspended in 190 μl of buffer solution. Then, 10 μl of propidium iodide (PI; 20 μg/mL) was added. The samples were then measured by flow cytometry (BD Accuri C6 flow cytometer; BD Biosciences).

The effect of DLGAP5-siRNA on the cell cycle was detected by flow cytometry with PI staining. The trypsin digestive cells were added to the 6-hole culture plate. The sample was centrifuged at 1000 rpm for 5 min after digestion. We discarded the supernatant liquid and re-suspended the cells with PBS. The sample was again centrifuged at 1000 rpm for 5 min. We discarded the supernatant liquid and fixed the sample with 75% alcohol. The temperature was kept at 4 °C overnight. The cells were washed twice with pre-cooled PBS. The supernatant liquid was then discarded, and 400 µl of PBS containing 50 µg/ml of PI and 100 µg/ml of RNA enzyme was added to the cell precipitate. The sample was incubated at 37 °C for 30 min. Flow cytometry was used to detect the cell cycle process under different processing conditions. WinMDI version software was used to analyze the cell cycle process.

#### Statistical analysis

All the obtained data came from 3 independent experiments and were expressed as mean ± standard deviation ($\bar{x}$ ± s). The statistical software SPSS 22.0 was used for inspection and analysis. TumGrowth, which is an open-access web tool for the statistical analysis of tumor growth curves, was used to compare cell growth [22]. Multigroup comparisons of the means were carried out by one-way analysis of variance (ANOVA) with post hoc contrasts by the Student–Newman–Keuls test. All means were calculated from at least 3 independent experiments. The statistical significance for all tests was set at P < 0.05.

## Results

### Screening differential genes using GEO data sets

**Fig. 1:**
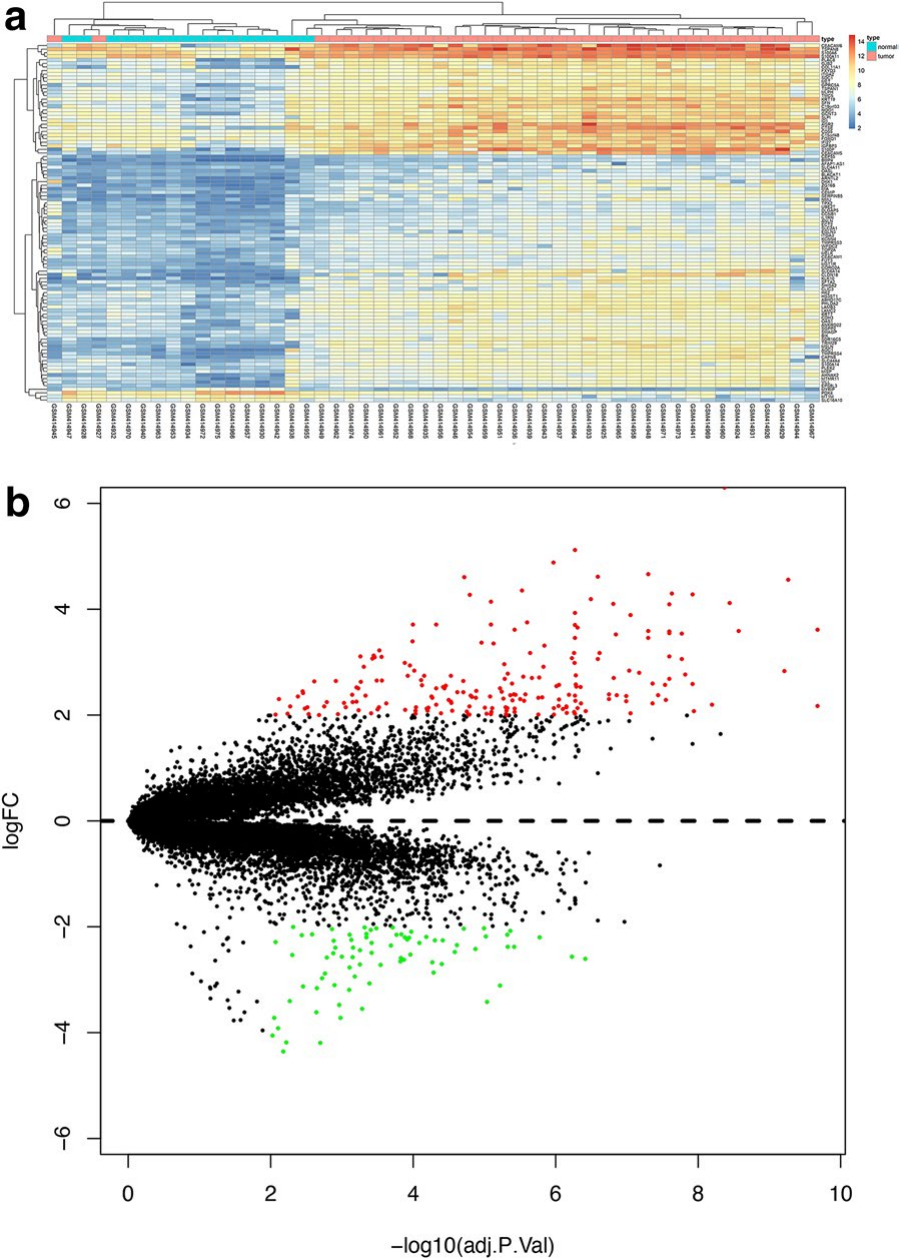
GEO differential gene heat map and volcano plot. **a** Heat map of differential gene expression. **b** Volcano plot of differential gene expression

We screened 201 significant upregulated differential genes and 79 downregulated genes (Additional file 1: Table S1), and chip correction was performed (Additional file 2: Figure S1). One hundred DEGs were selected to form a heat map, which is shown in Fig. 1a. Figure 1b is the volcano plot of the differential genes.

### Construction of the protein interaction network

**Fig. 2:**
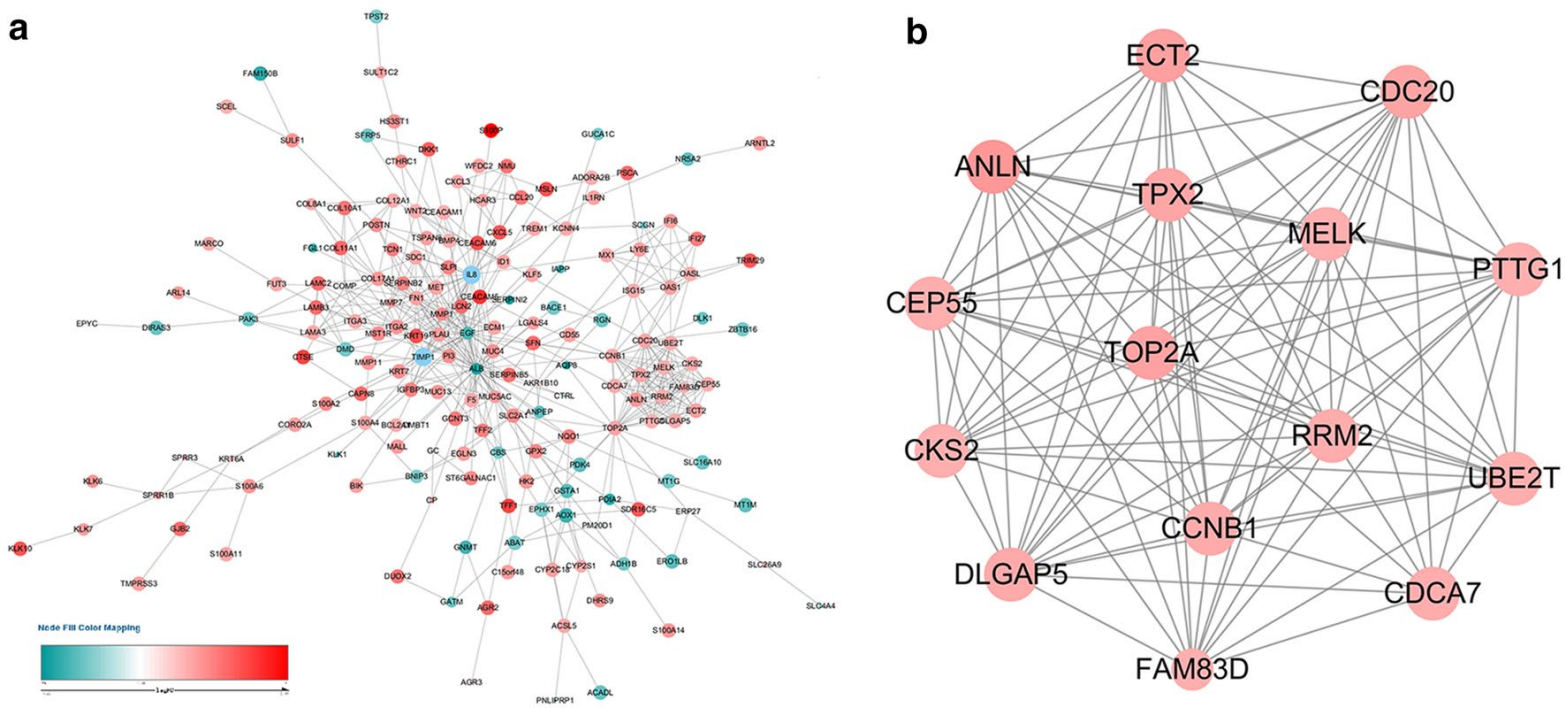
Construction of Protein Interaction Networks. **a** Differential gene protein interaction network. **b** Core module network

The structure of the differentially expressed genes’ protein interaction network is shown in Fig. 2a, which contained 15 nodes and 97 edges. It was obtained from the DEG PPI network using MCODE (Fig. 2b).

### Kaplan–Meier survival curves

**Fig. 3:**
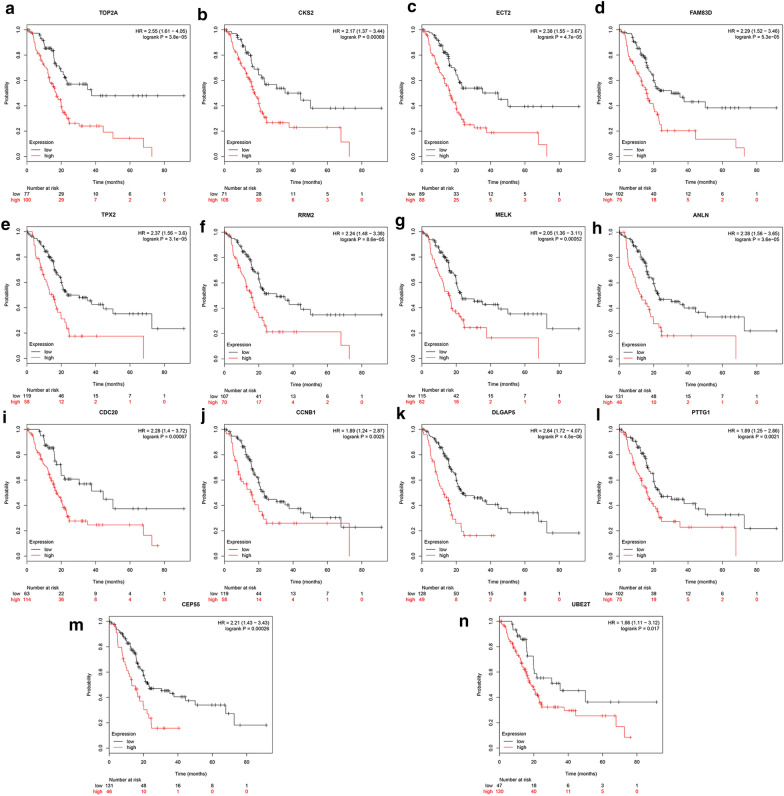
Analysis of the relationship between 14 genes and pancreatic cancer survival prognosis based on the Kaplan–Meier plotter database

The Kaplan–Meier Plotter database was used to analyze the survival prognoses of 15 new genes. Fourteen were related to pancreatic cancer prognosis. Their survival prognoses are shown in Fig. 3a–n. Among these genes, DLGAP5 had the greatest significance in terms of prognosis (HR = 2.64 [1.72–4.07], P = 4.5e−06). DLGAP5 was therefore chosen for further analysis (Fig. 3).

### Expression and prognosis analysis of DLGAP5

**Fig. 4:**
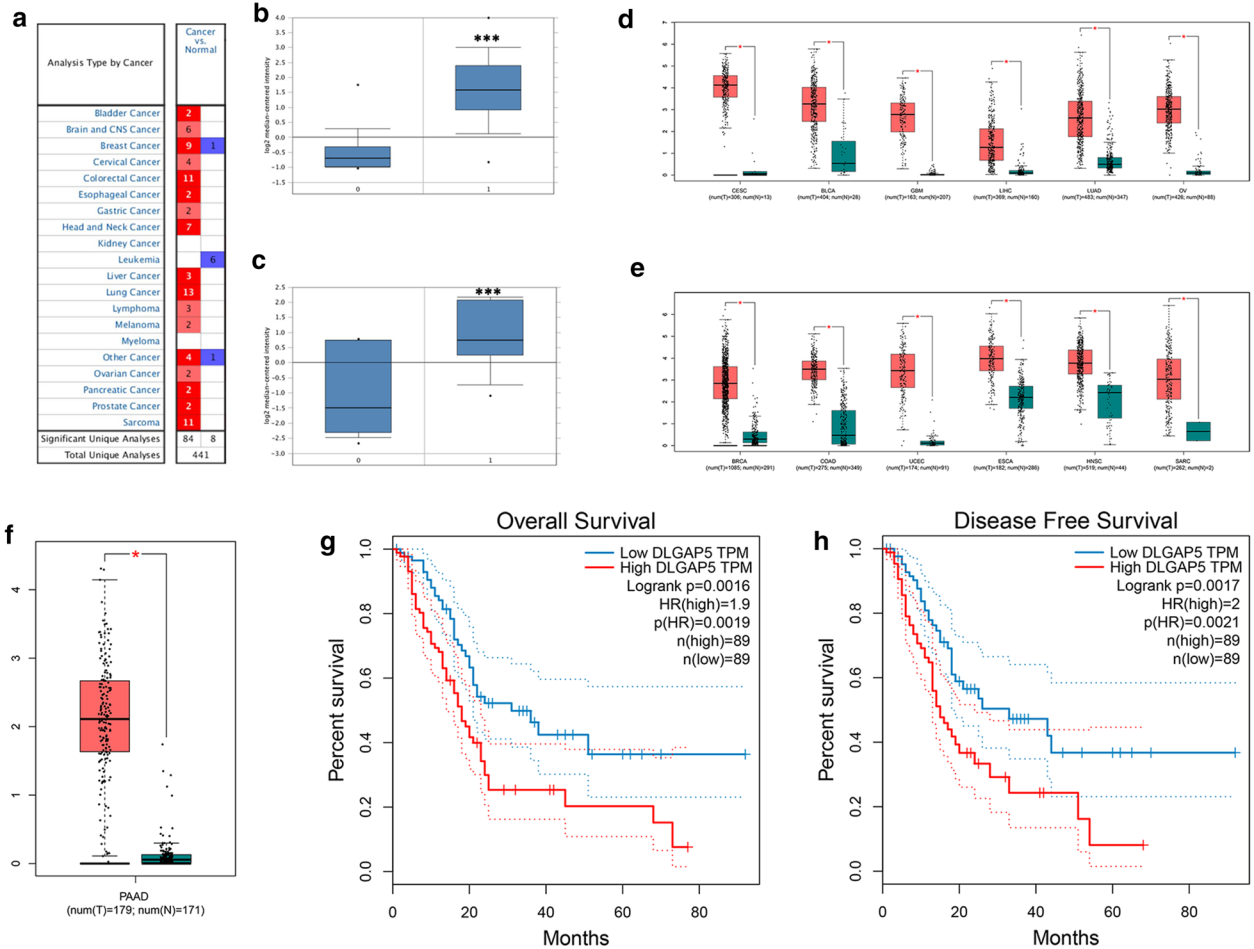
Differences in DLGAP5 expression and prognosis in oncomine. **a** DLGAP5 expression in all types of tumor in the Oncomine database. **b** DLGAP5 expression in the Grutzmann pancreatic cancer data set in Oncomine. **c** DLGAP5 expression in the Pei pancreatic cancer data set in Oncomine. **d**, **e** DLGAP5 expression in all types of tumor in the GEPIA database. **f** DLGAP5 differential expression in cancers and paracancers in the TCGA database. **g** The relationship between DLGAP5 expression and the overall survival of patients with pancreatic cancer. **h** The relationship between DLGAP5 expression and the progression-free survival of patients with pancreatic cancer

A total of 441 studies were collected from Oncomine (Fig. 4a). Among them, 92 showed a significant DLGAP5 expression change, with 84 showing increased expression and 8 showing decreased expression. Through the screening in Oncomine, 2 studies were found to involve DLGAP5’s expression in pancreatic cancer and normal tissues (Fig. 4b, c). In these studies, the expression level of DLGAP5 in the pancreatic cancer group was higher than in the normal group (P < 0.05) [28, 29]. The GEPIA database was then used to analyze DLGAP5’s expression in other tumors. Figure 4d, e shows the expression of DLGAP5 in 12 other cancers. DLGAP5 was generally highly expressed in many other cancers, thus playing an important role in the incidence and development of tumors. Figure 4f shows that DLGAP5 was significantly expressed in the TCGA data set of pancreatic cancer, and DLGAP5 expression levels had a great effect on patients’ overall survival (OS) and progression-free survival (PFS) (Fig. 4g, h). Compared with the low expression group, patients with pancreatic cancer in the high expression group had much shorter OS and PFS, indicating that the DLGAP5 expression is related to the OS and disease-free survival of patients with pancreatic cancer.

### The molecular mechanisms of DLGAP5

**Fig. 5:**
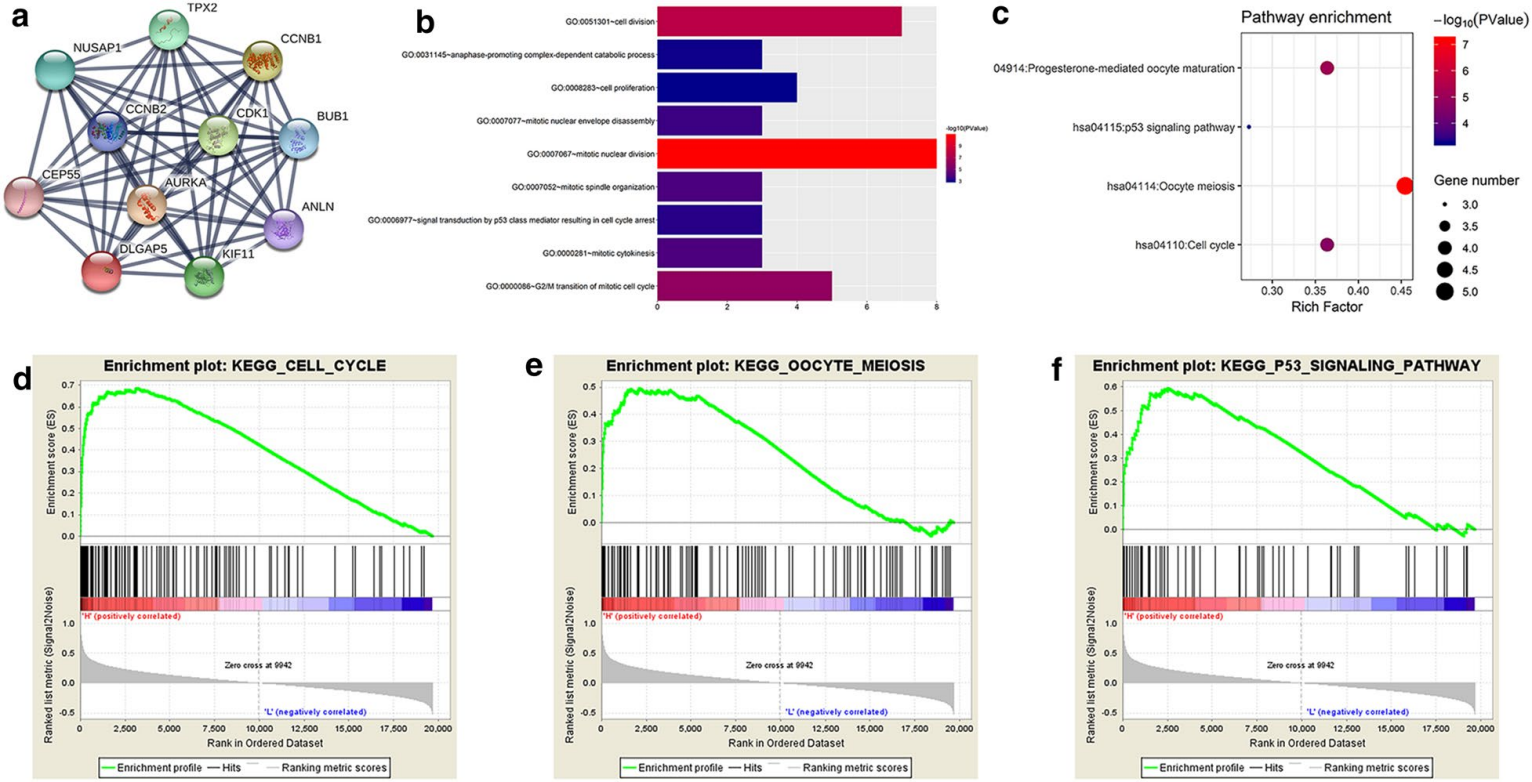
Molecular mechanism of DLGAP5’s involvement in pancreatic cancer incidence and development. **a** Protein interaction network constructed with DLGAP5-related molecules. **b** Functional bubble chart of DLGAP5-related molecules. **c** Bar graph of DLGAP5-related molecular pathways. **d**–**f** Pathways involved in the DLGAP5 enrichment analysis

The DLGAP5-related genes were found in the STRING database, a protein interaction network was constructed (Fig. 5a) for the functional and pathway enrichment analyses of DLGAP5-related molecules, and GO terms were visualized using the ggplot2 R software package. The genes were mainly enriched in biological activities such as protein kinase activity, mitotic nuclear division, cell division, and the G2/M transition of the mitotic cell cycle (Fig. 5b). The pathways involved mainly included oocyte meiosis, the cell cycle, and the p53 signaling pathway (Fig. 5c). The GSEA results suggest that DLGAP5’s high expression is significantly enriched into the cell cycle, p53 signaling pathway, oocyte meiosis (Fig. 5d–f), and other signaling pathways. This result is consistent with that from the DAVID database. It is believed that DLGAP5 may control tumor-related signaling pathways such as the cell cycle to pose influence on cell proliferation and then promote the incidence and development of pancreatic cancer.

### In vitro knockdown of DLGAP5 inhibited pancreatic cancer cell proliferation

**Fig. 6:**
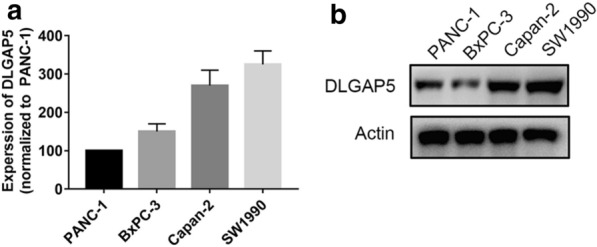
PCR and western blot verification of DLGAP5 expression in the pancreatic cancer cell lines. **a** PCR detection of the mRNA expression of DLGAP5 in the 4 pancreatic cancer cell lines. **b** Western blot verification of the protein expression of DLGAP5 in the 4 pancreatic cancer cell lines

**Fig. 7:**
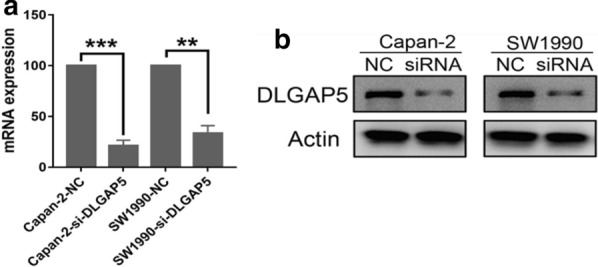
PCR and western blot verification of the knockdown efficiency of DLGAP5-siRNA. PCR (**a**) and Western blot (**b**) verifying that siRNA-mediated DLGAP5 inhibits the transfection efficiency of expression in Capan-2 and SW1990 cells. *P < 0.05, **P < 0.01, and ***P < 0.001

**Fig. 8:**
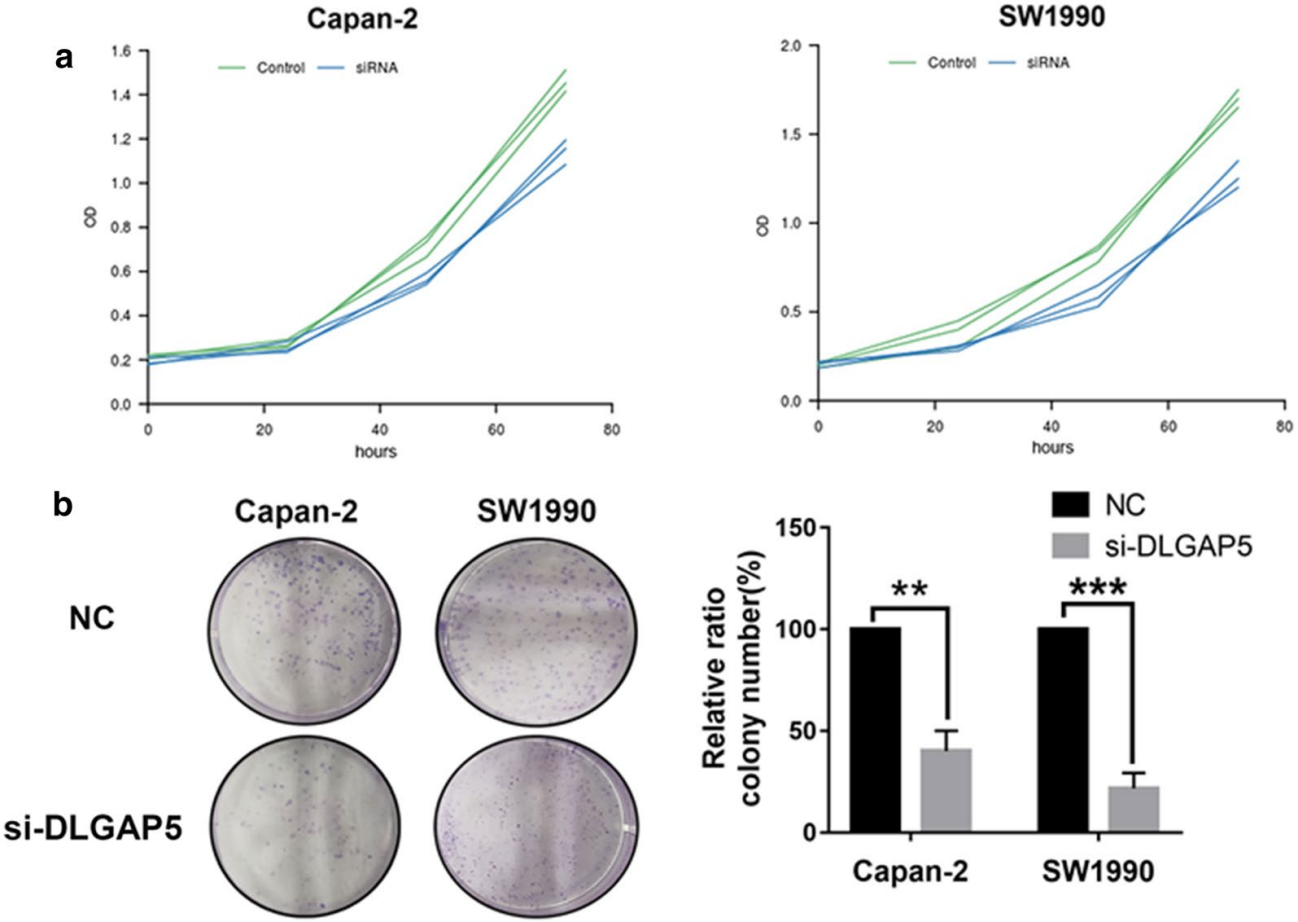
Effect of DLGAP5 on Pancreatic Cancer Cell Proliferation. MTT experiment (**a**) and colony formation test (**b**) detecting cell proliferation ability changes in Capan-2 and SW1990 cells after DLGAP5-siRNA transfection. In part A, P < 0.05; in part B, **P < 0.01 and ***P < 0.001

**Fig. 9:**
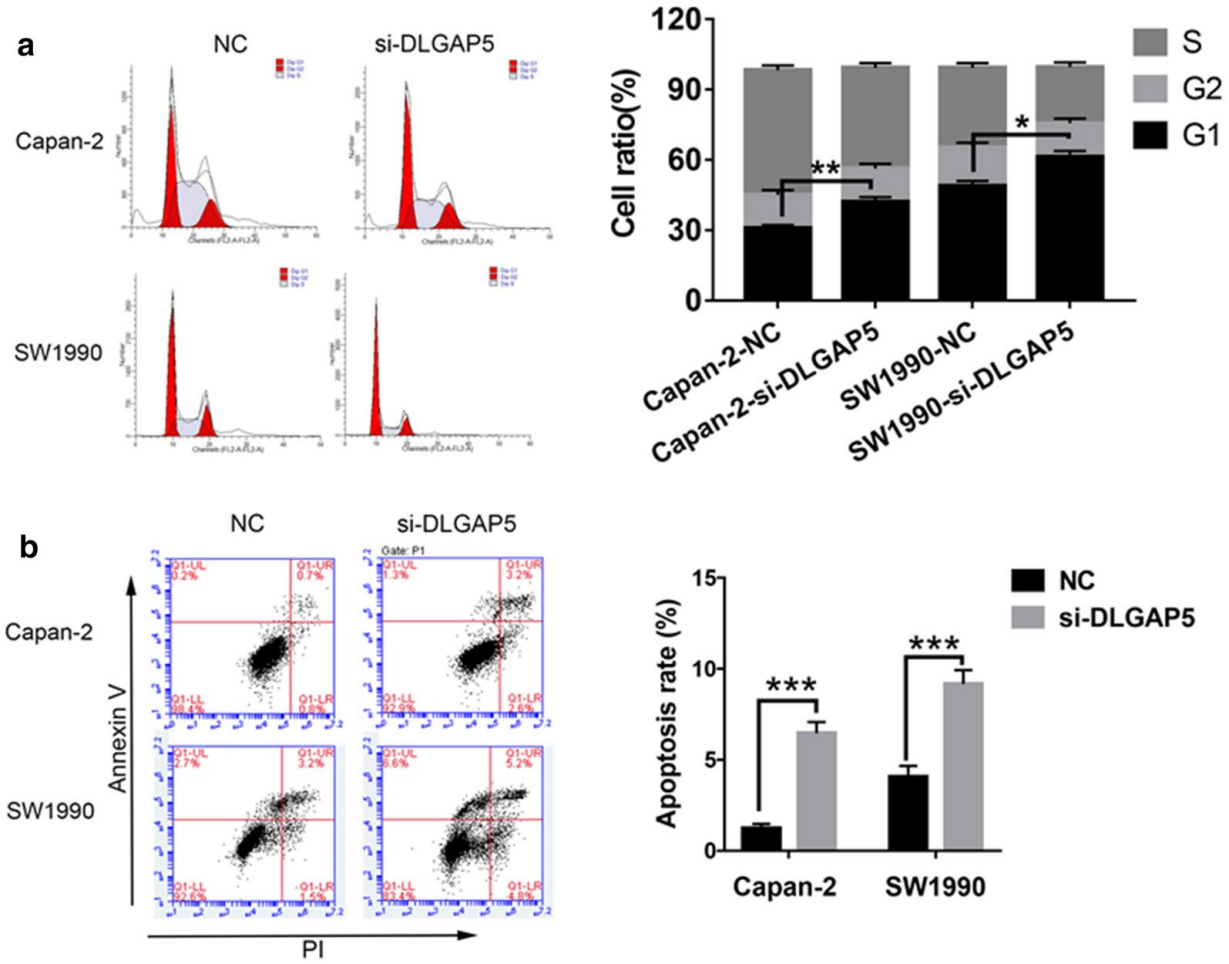
Effects of DLGAP5 on the cell cycle and apoptosis of pancreatic cancer cells. Flow cytometry detecting cell cycle (**a**) and apoptosis ability (**b**) changes in Capan-2 and SW1990 cells after DLGAP5-siRNA transfection. *P < 0.05, **P < 0.01, and ***P < 0.001

To select the appropriate cell model for the next study, we first compared the expression levels of DLGAP5 in pancreatic cancer cells (PANC-1, SW1990, Capan-2, and BxPC-3) (Fig. 6). The Capan-2 and SW1990 cell lines were selected for further study because the expression levels of DLGAP5 in SW1990 and Capan-2 cells were highest. The knockdown efficiency was evaluated by siRNA knockdown of DLGAP5, RT-qPCR, and Western blotting (Fig. 7). The MTT experiment showed that the proliferation ability of Capan-2 and SW1990 cells significantly decreased after DLGAP5 knockout (Fig. 8a). The colony formation experiment showed that there were significantly fewer colonies in the si-DLGAP5 group than in the si-NC group (Fig. 8b). The effect of DLGAP5 on the cell cycle was evaluated by flow cytometry, as shown in Fig. 9a. The cells had significant G1 arrest after DLGAP5 knockout. The effect of DLGAP5 on apoptosis was further evaluated through flow cytometry analysis. As shown in Fig. 9b, the apoptosis ability of the si-DLGAP5 transfection group was significantly enhanced. These results suggest that DLGAP5 knockout significantly inhibits pancreatic cancer cell proliferation.

### In vitro knockdown of DLGAP5 reduced pancreatic cancer cell invasion and migration

**Fig. 10:**
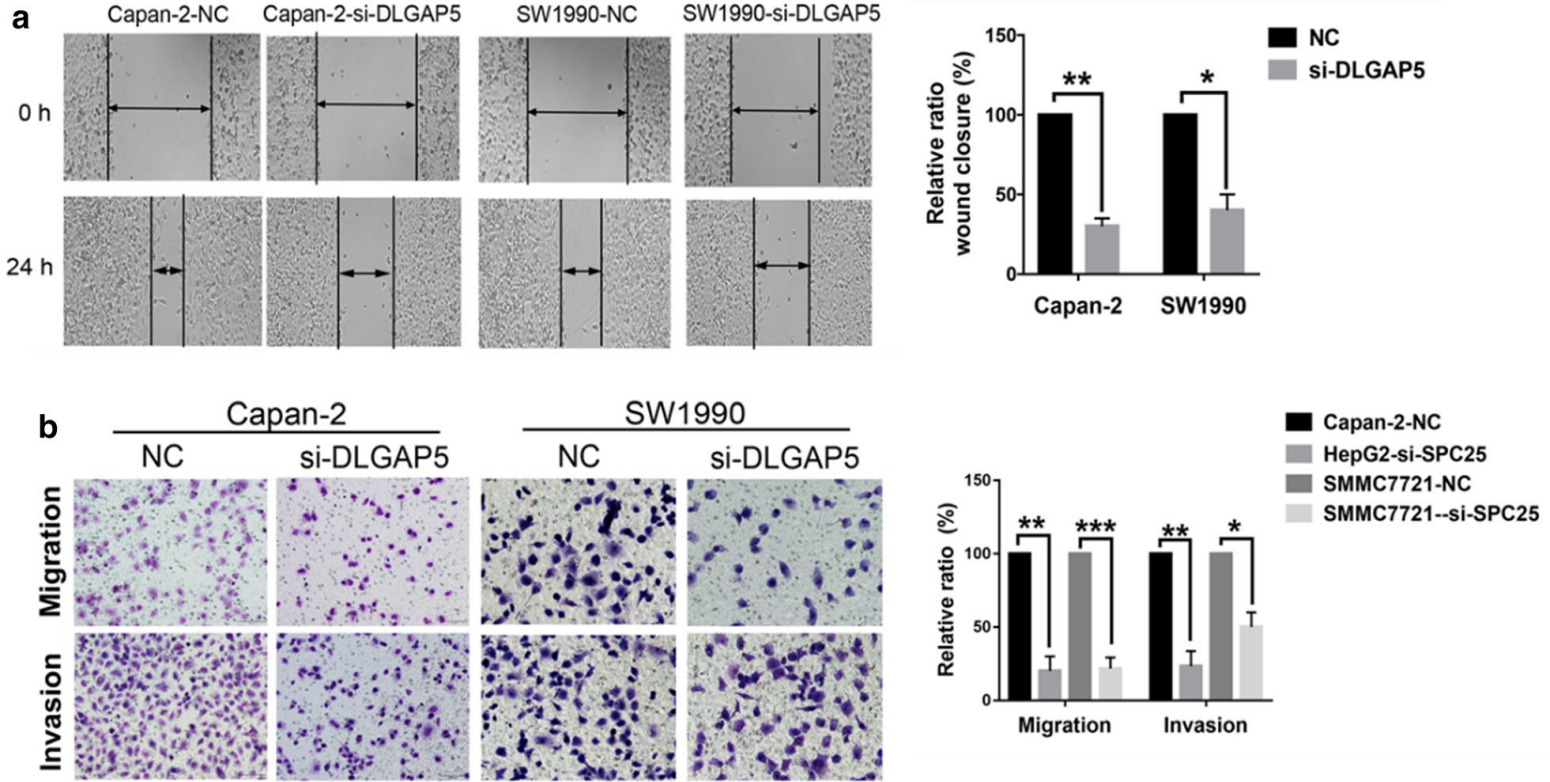
Effect of DLGAP5 on pancreatic cancer cell invasion, migration, and adhesion. **a** Scratch test showing that the knockdown of DLGAP5 could inhibit the migration ability of Capan-2 and SW1990 cells. **b** Forty-eight hours after DLGAP5-siRNA transfection, a Transwell experiment was performed. The numbers of cells on the Transwell membrane were compared, showing that migration and invasion ability significantly decreased. *P < 0.05, **P < 0.01, and ***P < 0.001

To evaluate the effects of DLGAP5 on the invasion and migration of pancreatic cancer cells, the wound healing experiment was performed, and it showed that DLGAP5 knockdown significantly inhibited the migration of Capan-2 and SW1990 cells (Fig. 10a). This result was further verified by the Transwell experiment in that the migration and invasion ability of the si-DLGAP5 transgenic group was significantly inhibited (Fig. 10b).

### Activation of the p53 pathway after in vitro knockout of DLGAP5

**Fig. 11:**
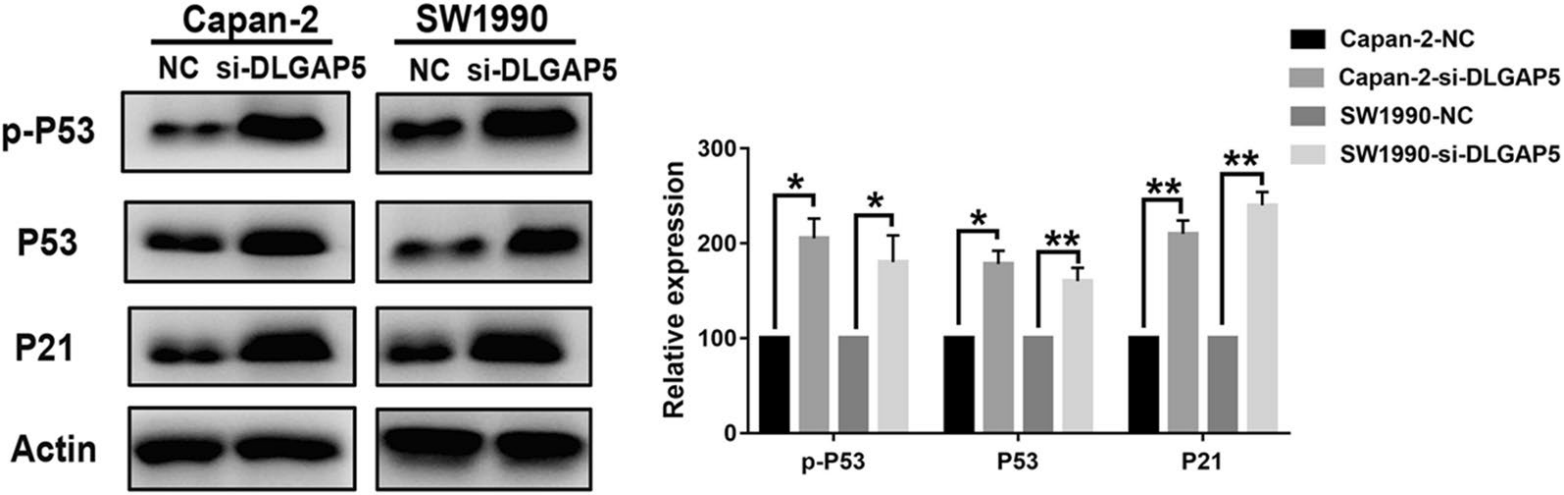
P53 pathway activation after knockout of DLGAP5. Protein expression changes of p53, p-p53, and p21 detected by Western blotting

We explored the relationship between DLGAP5 and the p53 pathway in pancreatic cancer. GSEA results showed a significant correlation between DLGAP5 and the p53 pathway, as verified by Western blotting. Figure 11 shows that the protein expression levels of p53, p-p53, and p21 significantly increased after DLGAP5 knockout in Capan-2 and SW1990 cells, suggesting that the p53 pathway is activated in pancreatic cancer cells after DLGAP5 knockout, which may inhibit the malignant phenotypes of pancreatic cancer cells.

## Discussion

With the wide application of genome-wide gene expression chips, many molecular markers have been developed, and gene expression profiles have provided important auxiliary means for predicting cancer prognosis. However, few factors significantly related to pancreatic cancer prognosis have been identified. This study aimed to combine bioinformatics and basic experiments to screen out genes with potential for pancreatic cancer prognosis. We screened out some genes with significant differences in the GEO database, performed a functional enrichment analysis, and constructed a protein interaction network of these differential genes. To screen out the genes closely related to the prognosis of pancreatic cancer from these differential genes, we used Kaplan–Meier to plot the survival curves of these differential genes and found that among these differential genes, the prognosis of the DLGAP5 gene was most significant. We believe that DLGAP5 is most closely associated to pancreatic cancer survival and can be used as a prognostic marker for pancreatic cancer. The biological role of DLGAP5 in pancreatic cancer prognosis is currently unclear; therefore, it was chosen for further analysis.

At present, many reports have been published on the biological function of the DLGAP5 gene in the occurrence and development of cancer. Schneider et al. studied the biological function of DLGAP5 in non-small cell lung cancer; through RT-qPCR and immunofluorescence, it was shown that DLGAP5 is more highly expressed in lung cancer tissues than in normal lung tissues and is associated with poor prognosis [20]. Shi et al. reached the same conclusion through the TCGA data set. In addition, the receiver operating characteristic (ROC) analysis and evaluation showed that DLGAP5 expression in patients with lung cancer and normal controls was significantly different. Kaplan–Meier analysis showed that increased expression of DLGAP5 was negatively correlated with OS and recurrence-free survival [19]. The above research showed that DLGAP5 can be used as a prognostic molecule for patients with lung cancer. Liao et al. showed that DLGAP5 expression is regulated by methylation and that its upregulation can promote the proliferation of hepatocellular tumors by promoting cell proliferation [23]. Horning et al. found the upregulation of DLGAP5 expression to be associated with recurrent prostate cancer [24]. DLGAP5 can stabilize spindle formation, leading to survival despite a microtubule challenge of docetaxel in androgen-regulated prostate cancer cell cycle systems [25]. DLGAP5 is also a direct downstream target of NOTCH3, which partially explains the mechanism of how NOTCH3 activation promotes ovarian cancer from the perspective of mitotic aberrations [26].

To the best of our knowledge, the role of DLGAP5 in pancreatic cancer has not yet been reported. To further clarify the molecular mechanisms of DLGAP5 in pancreatic cancer, we performed functional experiments on DLGAP5 in Capan-2 and SW1990 cells to study the involvement of DLGAP5 in biological behavior. MTT experiments showed that Capan-2 and SW1990 cell proliferation was significantly reduced after DLGAP5 was knocked out. Colony formation experiments showed that the number of colonies in the si-DLGAP5-transfected group was significantly less than that in the control group. After DLGAP5 was knocked out, the cells had a significant G1 phase arrest, and the si-DLGAP5 transfection group had significantly increased apoptosis ability. The above results indicate that DLGAP5 knockout significantly inhibited the proliferation of pancreatic cancer cells. Further, wound healing experiments showed that DLGAP5 knockdown could significantly inhibit the migration capacity of Capan-2 and SW1990 cells. Transwell experiments further verified this result; the migration and invasion ability of the si-DLGAP5-transfected group was significantly inhibited.

We then performed a pathway enrichment analysis on DLGAP5-related genes, and the results showed that the genes were mainly concentrated in protein kinase activity, mitotic nuclear division, cell division, the G2/M transition of the mitotic cell cycle, the p53 signaling pathway, and so on. DLGAP5 was significantly related to the p53 pathway. After we knocked out DLGAP5 in Capan-2 and SW1990 cells, the protein expression levels of p53, p-p53, and p21 were significantly increased. The above results indicate that DLGAP5 knockout activates the p53 pathway in pancreatic cancer cells. We speculate that DLGAP5 may inhibit the malignant phenotype of tumor cells through this pathway. The advantage of this study is that the expression and biological behavior changes of DLGAP5 in pancreatic cancer are discussed for the first time using a combination of bioinformatics analysis and experiments.

Protein kinases are enzymes that catalyze the transfer of phosphate from ATP to serine/threonine or tyrosine residues of target molecules. They play a key role in many aspects of cell function, including control of metabolism, transcription, cell division and movement, and programmability. Cells die and are involved in the immune response and nervous system function [27]. Protein kinases have conserved catalytic domains that phosphorylate protein substrates, and they thus play a key role in cell signaling pathways [28, 29]. Abnormal activation or regulation of protein kinases is a major cause of human disease, especially cancer [30]. In many cancers, mutations or abnormal expression of protein kinases are associated with tumorigenesis, metastasis, and resistance to chemotherapy. Tumor-associated protein kinases have become important molecular targets and biomarkers [31]. During mitosis, mitotic checkpoint defects may lead to chromosomal cohesion defects that cause sister chromatids to be erroneously separated, and centrosome amplification that promotes multipolar mitosis can lead to a loss or increase of chromosomes [32]. Chromosome error separation may lead to chromosome instability and aneuploidy, and mitotic defects or failures may lead to the production of aneuploid or tetraploid cells, which are the key markers of tumor cells [33–35]. Cell cycle disorders are the basis for the abnormal cell proliferation that characterizes cancer, and loss of cell cycle checkpoint control promotes genetic instability [36]. The complex mechanism of a cell cycle checkpoint includes sensors that monitor the integrity of a specific task and response elements that trigger the next downstream event, which will involve the actual process of DNA replication and isolation [37]. The transcription factor p53 plays an important role in the cell cycle and is the most important tumor suppressor [38]. After cellular stress signals, such as DNA damage or oncogenic stress, p53 is activated through a series of phosphorylation events and other post-translational modifications, followed by the expression of p53 target genes involved in cell cycle arrest, DNA repair, or apoptosis [39]. Perturbations in the p53 signaling pathway are considered necessary for most cancer developments, and there is evidence that the restoration or reactivation of p53 function will have significant benefits [40]. Although our results showed that DLGAP5 is associated with the p53 signaling pathway, there is still a lack of evidence that DLGAP5 is a useful marker only in p53-proficient pancreatic cancer cases. Our research has certain limitations that are similar to those of other studies. Although validated in databases and cell experiments, we lacked relevant animal experiments and reliable prospective clinical data. In addition, although our research identified some mechanisms that may be relevant to pancreatic cancer prognosis to some extent, further investigation of these mechanisms is needed.

## Conclusion

In this study, we screened out genes that were differentially expressed in pancreatic cancer and selected from them DLGAP5, which may be closely related to prognosis. The results of this study may help to predict the prognoses of patients with pancreatic cancer in the future.

## Supplementary information

**Additional file 1: Identification of differentlly expressed genes.**

**Additional file 2: Chip correction in GSE16515.**

## Data Availability

The datasets used and/or analyzed during the current study are available from the corresponding author on reasonable request.
