# Supplementary figures and images for: Bioinformatics analysis combined with experiments to explore potential prognostic factors for pancreatic cancer
Source: Cancer Cell Int. 2020 Aug 8;20:382. doi: 10.1186/s12935-020-01474-7 (PMC7414559; doi:10.1186/s12935-020-01474-7)

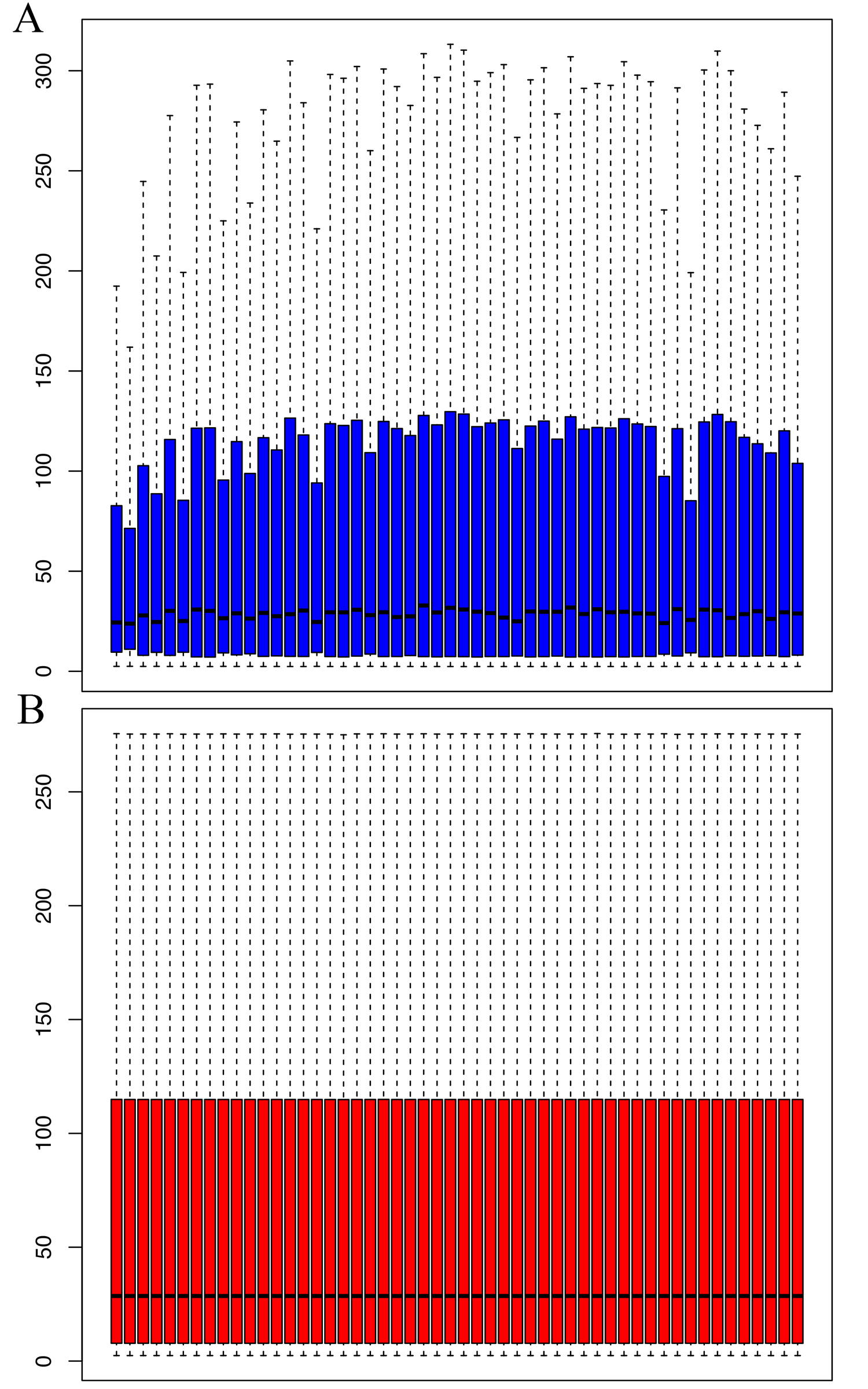

Supplement: Supplementary file 2 — Additional file 2: Chip correction in GSE16515. [file 12935_2020_1474_MOESM2_ESM.tif]
